# Brain abscess a grave complication of tetralogy of fallot: a rare clinical image

**DOI:** 10.11604/pamj.2022.42.82.35266

**Published:** 2022-05-31

**Authors:** Arjun Jaiswal, Amar Taksande

**Affiliations:** 1 Department of Pediatrics, Jawaharlal Nehru Medical College, Acharya Vinoba Bhave Rural Hospital, Datta Meghe Institute of Medical Sciences, Sawangi, Wardha, Maharashtra, India

**Keywords:** Brain abscess, seizure, tachycardia

## Image in medicine

A three-year-old female child presented to our Outpatient Department (OPD) with complaints of multiple episodes of seizure in one day. On examination, she had tachycardia and tachypnea; saturation was 60 % to 76% in all four limbs, Deep Tendon Reflex (DTR) was exaggerated, plantar jerks were extensor, central cyanosis, and grade III clubbing, and systolic ejection murmur on auscultation. She had no previous history of dyspnea and seizure. A complete blood count was suggestive of a white blood count of 17300 with neutrophilic predominance. A 2D echo done was suggestive of tetralogy of fallot. The patient was planned for magnetic resonance imaging of the brain. MRI brain was suggestive of a multi-located lesion in the left frontotemporal lobe suggestive of cerebral abscess, causing a midline shift of 4mm to the right side. The patient was started on ceftriaxone at 75mg/kg/day, amikacin at 15mg/kg/day and injection leverage at 20mg/kg/day, propranolol at 2mg/kg/day. A neurosurgery opinion was taken. She was planned and taken for surgery. Left frontal craniotomy was done with excision of the abscess wall. Histopathological examination confirmed the diagnosis of brain abscess. She was kept in PICU post-surgery, and antibiotics were continued for 14 days. Post-surgery she had no new episode of seizure. She was planned for cardiac surgery 1 and half months after craniotomy (A, B, C).

**Figure 1:**
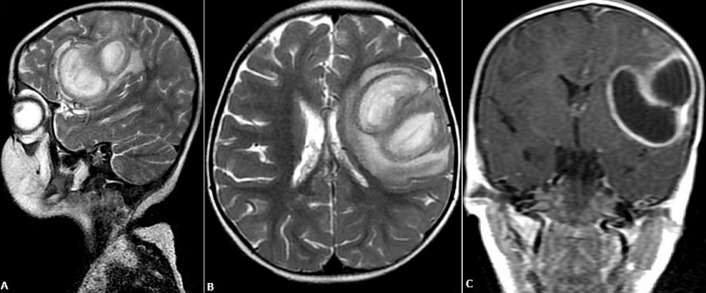
A) MRI brain (sagittal section) of head suggestive of mass in the front-tempero-parietal region; B) MRI brain (axial section) of brain suggestive of mass in the left lobe of the brain causing mass effect by doing a midline shift of 4mm; C) MRI brain (coronal section) showing a mass on the left side

